# The effect of a complex intervention for older adults on medication adequacy: results from the + AGIL Barcelona program

**DOI:** 10.1186/s12877-026-07699-x

**Published:** 2026-06-02

**Authors:** Francesco Salis, Ana María de Andrés-Lázaro, María Pamela Burbano, Luis Ignacio Soto-Bagaria, Francisco Díaz-Gallego, Neus Gual-Tarrada, Marco Inzitari, Laura Mónica Pérez-Bazán

**Affiliations:** 1 RE-FiT Barcelona Research Group, Vall d’Hebrón Institute of Research (VHIR), Barcelona, Catalunya Spain; 2 Department of Medical Sciences and Public Health, University of Cagliari, Cagliari, Italy; 3 Parc Sanitari Pere Virgili, Barcelona, Catalunya Spain; 4 Cap Raval Sud, Institut Catalá de La Salut, Barcelona, Catalunya Spain; 5 Geriatric Department, Vall d’Hebrón University Hospital, Barcelona, Catalunya Spain

**Keywords:** Frailty, Integrated care, Multidimensional, Pharmacology, Polypharmacy, Prescription

## Abstract

**Background:**

Frailty is a multidimensional syndrome associated with increased vulnerability to stressors, chronic disease exacerbations, hospitalizations, and disability. Polypharmacy, often defined as the chronic use of five or more medications, is closely and bidirectionally related to frailty. In this context, medication review is a potential strategy to improve health outcomes and slow down the frailty process by optimizing pharmacological treatments. This study aims to evaluate the impact of a geriatrician-led medication review, integrated within primary care (+ AGIL Barcelona program) on polypharmacy and medication adequacy in older adults.

**Methods:**

We evaluated the effect of the + AGIL Barcelona program on medication adequacy. The + AGIL is a multidisciplinary and multicomponent intervention aimed at promoting healthy aging, based on a comprehensive geriatric assessment and a 10–week structured exercise program, nutritional counseling, medication review, and healthy lifestyle promotion tailored to the individual's needs. Based on patient’s frailty status, comorbidities, and preferences, geriatricians revised the medications, and the modifications were discussed with the participants’ primary care physician, and in selected cases, with a clinical pharmacist. Baseline and three-month follow-up data were collected, including quantitative polypharmacy prevalence, and potentially inappropriate medications (PIMs), according to the EU(7)-PIM list. Subgroup analyses were based on Clinical Frailty Scale scores.

**Results:**

The study included 173 community-dwelling older adults (mean age: 81.2 years – SD: 5.7; 68.8% women). At baseline, polypharmacy was prevalent (84.4%), with 59.5% of participants receiving at least one PIM. After three months, a slight reduction in the mean number of medications per patient was observed (7.6 to 7.4, *p* = 0.051). Reduction of number of PIMs was also observed (1.0 to 0.9 per patient, *p* = 0.011), particularly in those medications affecting the nervous (from 30.1% to 24.3% in the sample, *p* = 0.025) and genitourinary (from 6.9% to 4.6%, *p* = 0.046) systems. After stratifying by frailty, a consistent trend was observed overall and especially among non-frail and vulnerable groups.

**Conclusions:**

While the + AGIL Barcelona program had a limited quantitative impact on polypharmacy, it significantly reduced PIMs’ use, particularly in nervous system-acting medications, which are involved in increased risk of falling and cognitive impairment in older adults. The study highlights the importance of medication review in multidimensional frailty management, although future research with larger samples and standardized medication review protocols is needed.

## Introduction

It is estimated that, by 2050, the number of people aged 65 years and older will double, exceeding 20% of the global population [34]. Accordingly, an increase in the prevalence of frailty is expected. Frailty is commonly described as a clinical syndrome characterized by increased vulnerability to endogenous and external stressors, reduced adaptability and heightened risk of adverse health outcomes [4]. Different studies across diverse settings and populations have demonstrated that targeted, multidomain interventions can prevent, delay or even revert frailty trajectories [14, 21, 33]. Accordingly, current scientific evidence and clinical practice advocate for a multidimensional approach with a comprehensive geriatric assessment (CGA) as the cornerstone of frailty management, enabling the identification of individual vulnerabilities and the design of person-centered interventions [4, 6, 23].

Among the domains commonly addressed within the CGA, polypharmacy and inappropriate prescriptions occupy a central role alongside cognitive impairment, functional decline, and multimorbidity [23]. Increasing attention has been directed toward qualitative aspects of medication use, particularly the appropriateness of prescriptions in relation to an individual’s clinical status, functional reserve, goals of care, and preferences [24]. A recent systematic review reported a prevalence of polypharmacy of 37% in the general population, rising to 45% among those aged 65 years and older [5]. Frailty and polypharmacy appear to be linked through a complex, bidirectional relationship [27], whereby frailty may increase susceptibility to medication-related harm, while inappropriate pharmacotherapy may accelerate functional decline and frailty progression. However, polypharmacy should be distinguished from inappropriate prescribing, as the use of multiple medications may also be clinically appropriate. In any case, the mentioned interplay underscores the need for multidimensional strategies that address both conditions simultaneously [7, 27].

Several tools are available to optimize medication use in older adults, including explicit prescribing criteria and lists of potentially inappropriate medications (PIMs) based on unfavorable benefit-risk profiles [18]. The need for a more personalized, goal-oriented approach to prescribing has become increasingly evident [2, 11]. Geriatricians play a pivotal role in this process, integrating CGA findings with clinical judgement to move beyond checklist-based recommendations and towards individualized medication management aligned with patients’ values and priorities. Medication review interventions have been shown to improve quality of life, facilitate deprescribing, and reduce hospital utilization, including hospital readmissions [2, 11].

Within this framework, the + AGIL Barcelona program was implemented in 2016 as a community-based, integrated care initiative targeting community-dwelling older adults in Barcelona, Spain [12, 25]. Designed as a real-world intervention, the program combines standardized multicomponent physical exercise, geriatric-lead medication review, and healthy lifestyle promotion. Previous evaluations have demonstrated its feasibility and effectiveness in promoting healthy aging, patient empowerment, and improving physical function, a surrogate marker of frailty [1, 25].

The aim of the present study is to assess the 3-month real-world effectiveness of the + AGIL Barcelona program on polypharmacy and medication adequacy among community-dwelling older adults, examining both quantitative and qualitative changes in pharmacotherapy and stratifying results by baseline frailty status.

## Methods

### Study design

This is a longitudinal, real-world implementation study conducted in primary care and community setting, evaluating the + AGIL Barcelona program between July 2016 to March 2020. The + AGIL Barcelona intervention was designed as a person-centered, multicomponent intervention co-designed with healthcare professionals, community stakeholders, and people participating in the program. It aims to promote healthy aging and address frailty through coordinating actions involving primary care professionals, geriatric teams, and local services. The present analysis focused on pharmacotherapy-related outcomes assessed at baseline and after three months of follow-up.

Given the pragmatic nature of the intervention and its integration into routine clinical practice, no control group was included. This design was chosen to reflect real-world conditions and feasibility, rather than to assess causal effects of controlled experimental settings.

### Description of the + AGIL Barcelona program intervention

The + AGIL Barcelona program is a multidimensional intervention based on CGA, used to identify individual needs and guide tailored care plans. Its implementation relied on the figure of a geriatrician and a physiotherapist, working weekly in the primary care center of Bordeta-Magòria (catsalut.gencat.cat). These professionals received referrals of potential candidate persons – older adults with preserved functional status and signs of frailty according to the Gérontopôle Frailty Screening Tool [32] -, performed a CGA and an individualized treatment plan including the following core components:

A) Multicomponent Exercise Program: participants attend a physiotherapist-led, 10-week multicomponent exercise program, consisting of 1-h weekly sessions. The program is tailored to each participant’s functional capacity. The exercise regimen includes resistance, endurance, balance, and flexibility training.
B) Lifestyle counseling: education and support to promote healthy behaviors, such as adherence to the Mediterranean diet, cessation of smoking, reduction or abstinence from alcohol use, and sleep hygiene, delivered by trained geriatricians through motivational interviewing techniques. In certain cases whose complexity required additional follow-up or monitoring, the patient's usual nursing team was also involved.
C) Comprehensive medication review: medication review is conducted as a part of the CGA by a geriatrician and informed by clinical judgement rather than a rigid deprescribing protocol. The process included a systematic evaluation of all chronic medications, considering indications, potential benefits and harms, drug-drug interactions, functional and cognitive status, life expectancy (based on frailty), and patient preferences. When appropriate, deprescribing or therapeutic modifications were proposed and discussed with the participants’ primary care physician, and in selected cases, with a clinical pharmacist. In detail, medication changes were proposed by the geriatricians based on the CGA. These recommendations were subsequently discussed with the patient’s primary care physician to reach a shared decision. In case of disagreement, decisions were resolved through direct communication between clinicians. Weekly, there was a time and space to hold meetings to reach consensus between geriatrician and primary care physician regarding treatment changes and other issues. If necessary, information was also exchanged by phone calls or emails. A clinical pharmacist was involved in selected cases with complex pharmacotherapy or high-risk medications. The geriatrician coordinated communication of final decisions and follow-up with participants in collaboration with primary care physicians. Medication changes were implemented collaboratively within routine care. All changes were documented in the patient’s clinical chart.

Within the Catalan health system, all citizens are assigned a primary care physician, a nurse, and an administrative support staff. The geriatricians involved in the + AGIL program worked directly within the primary care center and used shared health electronic records. A total of three geriatricians participated, all of whom had a solid background in geriatric pharmacotherapeutics. The program was designed to be adaptable to individual needs and sustainable over time, leveraging local healthcare and social care services, and digital tools. Further details regarding program development and implementation have been extensively described elsewhere [12, 25].

Participants were assessed at baseline and at 3 months. Although some participants continued within the broader intervention program beyond this period, only those who completed the 3-month follow-up assessment were considered for this longitudinal analysis.

### Study population

The study included community-dwelling older people identified as frail using the Gérontopôle Frailty Screening Tool [32] and referred to the program by their primary care team [25]. Eligible participants were aged 65 years and older. Exclusion criteria were primarily pragmatic and included inability to participate in the exercise component due to severe mobility limitations, acute illness, or logistical barriers.

### Variables and data collection

Baseline and follow-up assessments were conducted at program entry and 3-month follow-up. Sociodemographic characteristics (age, sex, living arrangement, marital status and educational level), comorbidity burden was assessed using the age-adjusted Charlson's Comorbidity Index [3].

Functional status was evaluated using the Barthel Index for basic activities of daily living [19] and the Lawton Index for instrumental activities of daily living [16], with higher scores indicating greater independence. Subjective health perception was recorded using a five-point Likert scale, ranging from “poor” to “excellent”. Frailty status was determined using the Clinical Frailty Scale (CFS), a validated global measure which classifies individuals from “very fit” to “terminally ill” [29]. For analytical purposes, participants were categorized as non-frail (CFS 1–3), vulnerable (CFS 4), or frail (CFS 5–8), based on baseline assessment.

Information on all regularly prescribed medications was collected at baseline and at three months through structured participant interviews and review of electronic health records. Medications were coded according to the Anatomical Therapeutic Chemical (ATC) classification system to ensure standardized reporting and facilitate subsequent analysis. PIMs were identified using the EU(7)-PIM list, which, together with anticholinergic burden, was used as pragmatic proxies for medication appropriateness within the context of this real-world intervention.

### Outcomes

The primary outcome was the change in pharmacotherapy over three months, assessed both quantitatively (number of medications – polypharmacy and hyperpolypharmacy) and qualitatively (presence and number of PIMs).

Secondary outcomes included specific therapeutic classes; special attention was given to medications associated with increased risks of falls, such as nervous system and genitourinary medications, and variation in anticholinergic burden (ACB) score before and after the intervention. It is an explicit tool that assigns a score to medications based on their anticholinergic activity, and the sum of the scores gives a total score for each participant. This approach has shown optimal quality to define anticholinergic burden [17].

Changes in the number of medications between baseline and follow-up were additionally categorized as increase, no change, or reduction to facilitate exploratory analyses of prescribing trajectories.

Quantitative pharmacotherapy outcomes included:

- Mean number of medications per participant
- Prevalence of polypharmacy (≥ 5 medications)
- Prevalence of hyperpolypharmacy (≥ 10 medications)

Qualitative pharmacotherapy outcomes focused on medication adequacy and included:

- Presence and number of PIMs, identified using the EU(7)-PIM list
- Distribution of PIMs by ATC therapeutic groups
- Use of medications associated with increased risk of falls, particularly those acting on the nervous and genitourinary systems
- Anticholinergic burden, assessed using the ACB scale

### Statistical analysis

Continuous variables were summarized as means and standard deviations (SD), while categorical variables were presented as absolute and relative frequencies. Comparison between baseline and post-intervention values were performed using the paired Student’s t-test for continuous variables, and the McNemar's test for categorical variables, as appropriate.

Subgroups analyses were conducted according to baseline frailty categories. An ordinal logistic regression model was used to explore the association between frailty status and changes in medication count, adjusting for age-adjusted comorbidity burden. The dependent variable was the direction of change in medication count, derived from the difference between baseline and follow-up medication numbers, and categorized as “reduction”, “no change”, and “increase”. These categories were treated as an ordinal outcome for analysis. Its results were expressed as odds ratios (ORs), and 95% confidence intervals (95%CIs).

All analyses were considered exploratory due to the pragmatic design and limited sample size, particularly within frailty subgroups. Statistical analyses were conducted using Stata 15 (StataCorp, College Station, TX, USA), and R Studio (version 4.2.3).

## Results

**Fig. 1:**
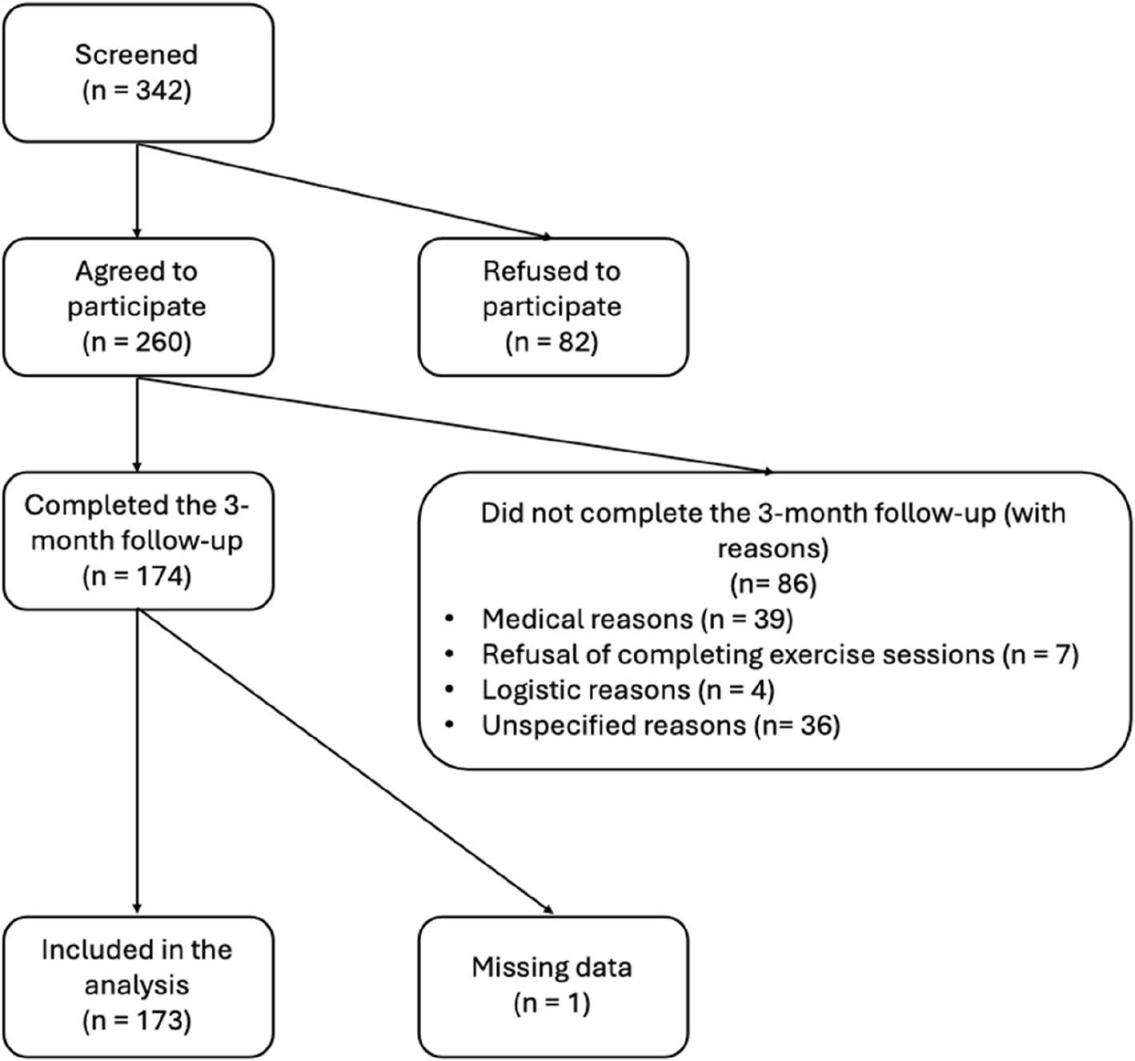
Flow-chart of included subjects

From an initial pool of 342 screened individuals, 260 (76.0%) agreed to participate in the program. At the time of analysis, 174 participants (66.9%) had completed the 3-month follow-up. One participant lacked complete pharmacotherapy data at follow-up and was excluded, resulting in a final analytical sample of 173 participants (see Fig. 1). Only individuals with complete baseline and three-month assessments were included in this longitudinal analysis. Reason for attrition (*n* = 86) was primarily due to medical reasons (*n* = 39, including 3 deaths), refusal of completing exercise sessions (*n* = 7), logistic reasons (*n* = 4), and unspecified reasons (*n* = 36).

A total of 173 community-dwelling older adults were included in the final analysis. The mean age was 81.2 years (SD = 5.7), and 68.8% of participants were women. Overall, the comorbidity burden was moderate, with a mean age-adjusted Charlson Comorbidity Index 5.4 (SD 1.6). According to baseline frailty status, 24.3% of participants were classified as non-frail (CFS 1–3), 43.3% as vulnerable (CFS 4), and 32.4% as frail (CFS 5–8). Functional capacity was generally preserved, with mean Barthel and Lawton Index scores of 91.0 (SD 11.8) and 5.3 (SD 2.6), respectively. More than two-thirds of participants rated their health as fair to good. Baseline sociodemographic and clinical characteristics stratified by frailty status are presented in Table 1.

**Table 1:** Baseline sociodemographic, clinical and functional characteristics of the enrolled subjects, stratified by frailty status

Variable	Whole sample *N* = 173	Non-frail *N* = 42	Vulnerable *N* = 75	Frail *N* = 56
Age (years), mean (SD)	81.2 (5.7)	80.3 (5.1)	80.7 (5.6)	82.7 (6.0)
Sex, females, n (%)	119 (68.8)	33 (78.6)	48 (64.0)	38 (67.9)
Living arrangement: living alone, n (%)	69 (39.9)	22 (52.4)	30 (40.0)	17 (30.4)
Marital status, n (%)				
Married	78 (45.1)	17 (40.5)	35 (46.2)	26 (46.4)
Divorced	5 (2.9)	1 (2.4)	3 (4.0)	1 (1.8)
Single	18 (10.4)	4 (9.5)	8 (10.3)	6 (10.7)
Widow/er	72 (41.6)	20 (47.6)	29 (39.5)	23 (41.1)
Education, n (%)				
Illiterate	7 (4.0)	1 (2.4)	2 (2.7)	7.1
Primary school	51 (29.5)	14 (33.3)	22 (29.3)	26.8
Secondary school	89 (51.5)	18 (42.9)	38 (50.7)	59.0
University degree	26 (15.0)	9 (21.4)	23 (17.3)	7.1
Age-adjusted Charlson Comorbidity Index, mean (SD)	5.4 (1.6)	5.0 (1.8)	5.2 (1.5)	5.8 (1.6)
Barthel Index, mean (SD)	91.0 (11.8)	96.2 (5.6)	94.7 (5.0)	82.0 (16.1)
Lawton Index, mean (SD)	5.3 (2.6)	6.9 (1.8)	5.9 (2.2)	3.3 (2.4)
Health self-assessment, n (%)				
Low	36 (20.8)	2 (4.7)	16 (21.3)	18 (32.1)
Fair	48 (27.8)	7 (16.7)	25 (33.3)	16 (28.6)
Average	32 (18.5)	12 (28.6)	14 (18.7)	6 (10.7)
Good	53 (30.6)	19 (45.2)	28 (24.0)	16 (28.6)
Excellent	4 (2.3)	2 (4.8)	2 (2.7)	0 (0.0)

Frailty groups defined according to baseline Clinical Frailty Scale (CFS) scores: non-frail (CFS 1–3), vulnerable (CFS 4), frail (CFS 5–8). Values are presented as mean (standard deviation, SD) or frequencies, as appropriate

### Quantitative pharmacotherapy outcomes

At baseline, participants were prescribed a mean of 7.6 (SD 3.4). Polypharmacy (≥ 5 medications) was highly prevalent, affecting 84.4% of the sample, while hyperpolypharmacy (≥ 10 medications) was observed in 27.2% of the participants.

After three months, a small reduction in the mean number of medications per participant was observed (7.6 at baseline vs 7.4 at follow-up, *p* = 0.051); however, this change did not reach statistical significance. Similar non-significant trends were observed across frailty sub-groups (Table 2). The prevalence of polypharmacy decreased from 84.4% to 83.2% (*p* = 0.564), and hyperpolypharmacy from 27.2% to 26.6% (*p* = 0.808). In the vulnerable group, a slight increase in the hyperpolypharmacy was observed (from 32.0 to 36.0%), through this change was not statistically significant (*p* = 0.317).

**Table 2:** Changes in quantitative polypharmacy indicators from baseline to three-month follow-up, overall and by frailty status

Variable	Whole sample *N* = 173			Non-frail *N* = 42			Vulnerable *N* = 75			Frail *N* = 56		
	t0	t1	*p*-value	t0	t1	*p*-value	t0	t1	*p*-value	t0	t1	*p*-value
N. medications per patient (mean, SD)	7.6 (3.4)	7.4 (3.2)	0.051	6.6 (3.1)	6.4 (3.1)	0.145	7.9 (3.5)	7.7 (3.3)	0.106	8.0 (3.5)	7.8 (3.0)	0.139
Polypharmacy (%)	84.4	83.2	0.564	81.0	78.6	0.564	86.7	84.0	0.414	83.9	85.7	0.564
Hyperpolypharmacy (%)	27.2	26.6	0.808	11.9	9.5	0.317	32.0	36.0	0.317	32.1	26.8	0.257

t0, baseline; t1, after intervention; polypharmacy, use of ≥ 5 medications; hyperpolypharmacy, ≥ 10 medications; frailty groups defined according to baseline Clinical Frailty Scale (CFS) scores: non-frail (CFS 1–3), vulnerable (CFS 4), frail (CFS 5–8). Values are presented as mean (standard deviation, SD) or frequencies, as appropriate

Changes in medications count should be interpreted within the context of medication optimization rather than numerical reduction alone

When changes in medication count were categorized, 62 participants experienced a reduction in the number of medications, 73 showed no change, and 38 experienced an increase. No significant associations were observed between baseline frailty status and direction of change in medication count, either before or after adjustment for age-adjusted comorbidity burden (see Table 3).

**Table 3:** Ordinal logistic regression analysis of factors associated with change in medication count

Model	Adjusted for	Comparison	OR	95%CI
Unadjusted	-	Non-frail vs vulnerable	1.07	0.53–2.16
		Non-frail vs frail	1.05	0.50–2.23
Adjusted	Age-adjusted Charlson’s comorbidity index	Non-frail vs vulnerable	1.06	0.52–2.14
		Non-frail vs frail	1.01	0.57–2.17

OR, odds ratio; 95%CI, 95% confidence interval; frailty groups defined according to baseline Clinical Frailty Scale (CFS) scores: non-frail (CFS 1–3), vulnerable (CFS 4), frail (CFS 5–8); ordinal variable defined as the difference between baseline and follow-up medication numbers: reduction, no change, increase

### Potentially inappropriate medications

At baseline, the mean number of PIMs per participant was 1.0 (SD 1.1). Overall, 59.5% of the participants were prescribed at least one PIM, 30.1% at least two PIMs, and 9.3% at least three PIMs.

**Fig. 2:**
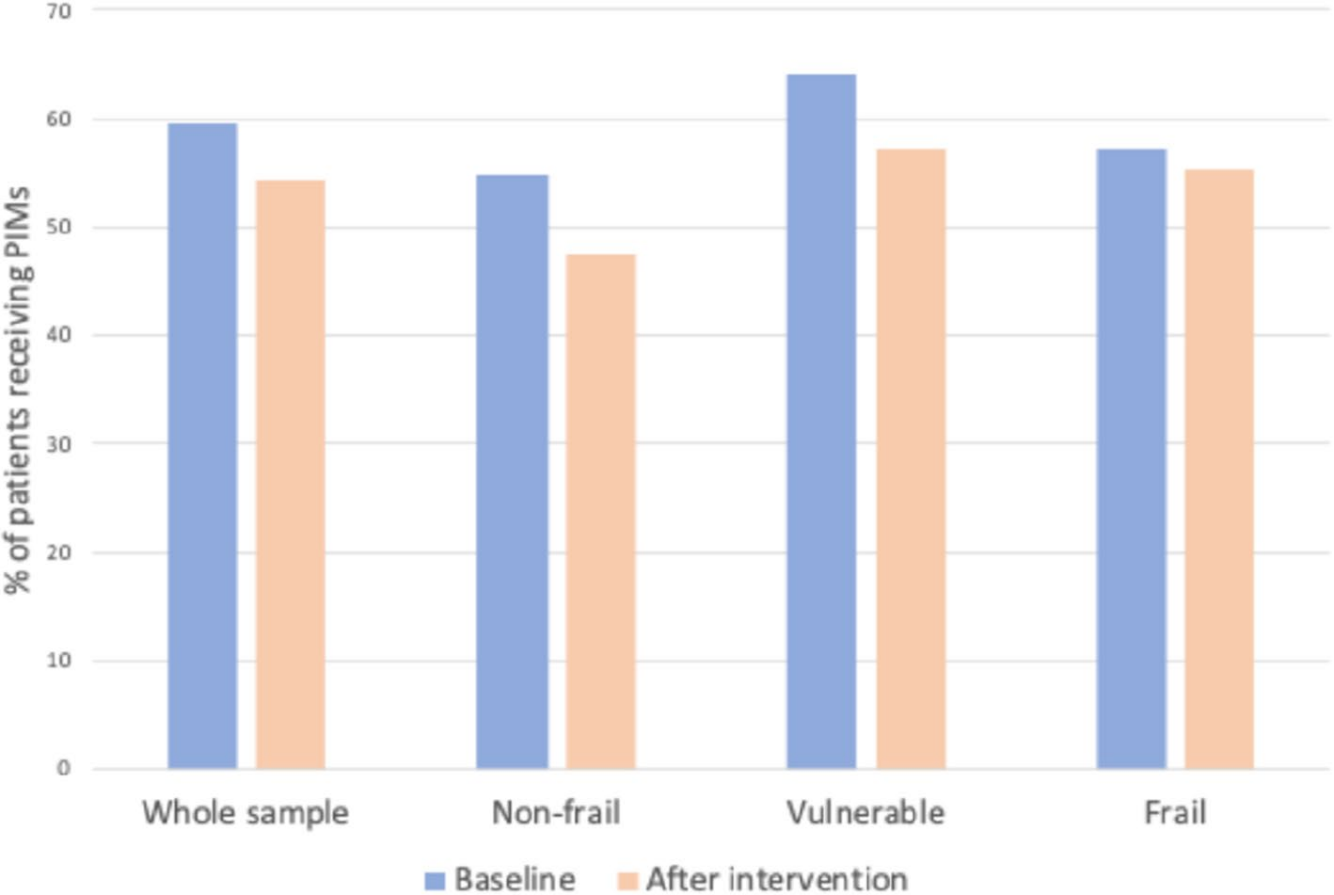
Changes in the prevalence of potentially inappropriate medications use by frailty status Percentage of patients receiving potentially inappropriate medications (PIMs) at baseline and three-month follow-up, stratified by frailty status. Frailty groups are defined according to baseline Clinical Frailty Scale (CFS) scores: non-frail (CFS 1–3), vulnerable (CFS 4), frail (CFS 5–8). Although absolute changes were small, consistent downward trends were observed across groups, reflecting real-world medication optimization

Following the intervention, a modest but statistically significant reduction in the mean number of PIMs per participant was observed (1.0 to 0.9, *p* = 0.011). Although reductions were observed across all frailty categories, these did not reach the statistical significance within individuals (Table 4 and Fig. 2). Similarly, the proportions of participants with at least one, two or three PIMs showed a consistent downward trend but did not differ significantly from baseline (see Table 4).

**Table 4:** Changes in the number and prevalence of potentially inappropriate medications from baseline to follow-up

Variable	Whole sample *N* = 173			Non-frail *N* = 42			Vulnerable *N* = 75			Frail *N* = 56		
	t0	t1	*p*-value	t0	t1	*p*-value	t0	t1	*p*-value	t0	t1	*p*-value
N. PIMs per patient (mean, SD)	1.0 (1.1)	0.9 (1.0)	0.011	0.9 (0.9)	0.7 (0.8)	0.109	1.1 (1.1)	1.0 (1.0)	0.068	1.0 (1.1)	1.0 (1.1)	0.242
Patients with ≥ 1 PIM (%)	59.5	54.3	0.061	54.8	47.6	0.317	64.0	57.3	0.096	57.1	55.4	0.656
Patients with ≥ 2 PIMs (%)	30.1	27.2	0.166	26.2	19.1	0.180	33.3	32.0	0.706	28.6	26.8	0.317
Patients with ≥ 3 PIMs (%)	9.3	7.0	0.157	4.8	2.4	0.317	12.0	8.0	0.180	8.9	8.9	1

t0, baseline; t1, after intervention; PIM, potentially inappropriate medication; frailty groups defined according to baseline Clinical Frailty Scale (CFS) scores: non-frail (CFS 1–3), vulnerable (CFS 4), frail (CFS 5–8). Values are presented as mean (standard deviation, SD) or frequencies, as appropriate

PIMs were identified using the EU(7)-PIM list and used as a proxy for medication adequacy

### Therapeutic classes of potentially inappropriate medications

The analysis by therapeutic groups showed significant reductions in the proportions of participants receiving PIMs acting on nervous system (30.1% to 24.3%, *p* = 0.025), and genitourinary system (6.9% to 4.6%, *p* = 0.046), as displayed in Table 5 (for some subgroups, *p*-values are not reported due to small sample sizes, which made statistical testing inappropriate). Reductions in these therapeutic classes were observed across frailty strata, although statistical significance was generally not reached in subgroup analyses. In frail participants, minimal changes in PIM prevalence were observed (see Table 5).

**Table 5:** Changes in potentially inappropriate medications by therapeutic class from baseline to follow-up

Variable (% patients)	Whole sample N = 173			Non-frail N = 42			Vulnerable N = 75			Frail N = 56		
	t0	t1	p-value	t0	t1	p-value	t0	t1	p-value	t0	t1	p-value
A. Alimentary tract and metabolism	4.6	3.5	0.158	4.8	4.8	1	6.7	4.0	0.1573	1.8	1.8	NP
B. Blood and blood forming organs	19.7	20.2	0.655	16.7	14.3	0.317	20.0	21.3	0.564	21.4	23.2	0.317
C. Cardiovascular system	22.0	22.0	1	11.9	14.3	0.564	24.0	24.0	1	26.8	25.0	1
G. Genitourinary system	6.9	4.6	0.046	9.5	4.8	0.157	6.7	4.0	0.157	5.4	5.4	0.317
M. Musculo-skeletal system	1.7	2.9	0.317	0.0	4.8	0.157	2.7	2.7	NP	1.8	1.8	NP
N. Nervous system	30.1	24.3	0.025	33.3	23.8	0.103	29.3	21.3	0.058	28.6	28.6	1
R. Respiratory system	1.7	1.7	NP	0.0	0.0	NP	2.7	2.7	NP	1.8	1.8	NP

t0, baseline; t1, after intervention; PIM, potentially inappropriate medication; frailty groups defined according to baseline Clinical Frailty Scale (CFS) scores: non-frail (CFS 1–3), vulnerable (CFS 4), frail (CFS 5–8); NP: p-value not provided (subgroup with insufficient sample size). Values are presented as frequencies

Therapeutic classes are based on the Anatomical Therapeutic Chemical (ATC) classification system. Nervous system and genitourinary medications are highlighted due to their association with falls, cognitive impairment, and other adverse outcomes in older adults

### Medications acting on the nervous and genitourinary systems

Further analysis of medications acting on the nervous system showed a significant overall reduction in the use of analgesics (57.2% to 51.5%, *p* = 0.003), and psycholeptics – including antipsychotics, anxiolytics, and hypnotic-sedatives (42.8% to 33.5%, *p* < 0.001) over the three-month period (see Table 6; for some subgroups, *p*-values are not reported due to small sample sizes, which made statistical testing inappropriate). Reduction in psycholeptics use was evident among non-frail and vulnerable participants. Conversely, an increase in the use of psychoanaleptics, mainly antidepressants, was observed among vulnerable participants (29.3% to 38.7%, *p* = 0.020). No significant changes were detected in the use of antiepileptics, antiparkinsonian drugs, or other nervous system medications.

**Table 6:** Changes in the use of nervous system medications from baseline to follow-up, overall and by frailty status

Variable (% patients)	Whole sample *N* = 173			Non-frail *N* = 42			Vulnerable *N* = 75			Frail *N* = 56		
	t0	t1	*p*-value	t0	t1	*p*-value	t0	t1	*p*-value	t0	t1	*p*-value
N01. Anesthetics	2.9	2.3	0.317	0.0	0.0	NP	4.0	4.0	1	3.6	1.8	0.317
N02. Analgesics	57.2	51.5	0.033	52.4	45.2	0.083	60.0	49.3	0.011	57.1	58.9	0.739
N03. Antiepileptics	11.0	13.3	0.103	0.0	0.0	NP	16.0	18.7	0.317	12.5	16.1	0.157
N04. Antiparkinson	4.6	4.1	0.317	2.4	2.4	NP	6.7	5.3	0.317	3.6	3.6	1
N05. Psycholeptics	42.8	33.5	0.000	45.2	33.3	0.025	45.3	32.0	0.002	37.5	35.7	0.655
N06. Psychoanaleptics	35.8	38.2	0.371	33.3	26.2	0.180	33.3	42.7	0.020	41.1	41.1	1
N07. Other	5.8	3.5	0.103	2.4	2.4	NP	8.0	4.0	0.083	5.4	3.6	0.564

t0, baseline; t1, after intervention; PIM, potentially inappropriate medication; frailty groups defined according to baseline Clinical Frailty Scale (CFS) scores: non-frail (CFS 1–3), vulnerable (CFS 4), frail (CFS 5–8); NP: p-value not provided (subgroup with insufficient sample size). Values are presented as frequencies

Therapeutic classes are based on the Anatomical Therapeutic Chemical (ATC) classification system – nervous system subclasses. Opposing trends in sedative-related drugs and antidepressants illustrate frailty-informed and symptom-oriented prescribing decisions

As for medications acting on the genitourinary system, a significant overall reduction was observed in the use of urologicals (20.8% to 12.7%, *p* = 0.018), especially among vulnerable patients (25.3% to 9.3%, *p* = 0.025). No other significant changes were detected in the use of other medications acting on the genitourinary system.

### Anticholinergic burden

At baseline, the mean ACB score was 1.0 (SD 1.6). After the intervention, a non-significant reduction was observed (mean 0.8, SD 1.4; *p* = 0.161). No significant changes were found in the distribution of participants across ACB scores categories, either in the overall sample or after stratification by frailty status (see Table 7).

**Table 7:** Changes in the anticholinergic burden from baseline to follow-up, overall and by frailty status

Variable	Whole sample *N* = 173			Non-frail *N* = 42			Vulnerable *N* = 75			Frail *N* = 56		
	t0	t1	*p*-value	t0	t1	*p*-value	t0	t1	*p*-value	t0	t1	*p*-value
ACB score 0 (%)	58.4	63.6	0.590	52.5	61.0	0.396	64.8	69.9	0.667	54.0	56.9	0.969
ACB score 1 (%)	21.1	21.2		20.0	26.8		16.9	16.4		28.0	23.5	
ACB score 2 (%)	11.2	7.3		10.0	4.9		12.7	6.8		10.0	9.8	
ACB score 3 or more (%)	9.3	7.9		17.5	7.3		5.6	6.8		8.0	9.8	

t0, baseline; t1, after intervention; ACB, anticholinergic burden; frailty groups defined according to baseline Clinical Frailty Scale (CFS) scores: non-frail (CFS 1–3), vulnerable (CFS 4), frail (CFS 5–8). Values are presented as frequencies

## Discussion

This real-world implementation study found that the + AGIL Barcelona program was associated to modest reductions in quantitative polypharmacy, alongside measurable improvements in qualitative aspects of pharmacotherapy, particularly reduction in PIMs. These results are consistent with the concept of appropriate polypharmacy, pointing out that medication optimization in older adults ought to prioritize benefit–risk balance, in accordance with individual care goals, rather than prioritizing only on reducing medication count.

Although polypharmacy was highly prevalent at baseline, the mean number of medications per participant decreased only slightly over the three-month follow-up, without reaching statistical significance. Comparable results have been observed in previous studies, especially among populations with multimorbidity and relatively stable baseline health [31, 35]. The primary aim of the + AGIL Barcelona program was not indiscriminately deprescribing, but rather the optimization of pharmacotherapy within the CGA framework [8, 10]. In this context, maintaining or even increasing the number of medications may be appropriate when treatments are tailored to patients’ clinical needs, burden of symptoms, and individual care goals.

This perspective, namely that medication optimization should prioritize tailored interventions, is especially relevant in the context of frailty-informed prescribing. Frailty should not necessarily be interpreted as a need for fewer medications, but rather as requiring more subtle prescribing decisions that deliberately consider potential benefits against risks. Preventive as well as symptom-oriented treatments may be appropriate across all levels of frailty when clinically indicated, while it has been shown that especially in more advanced frailty, pharmacotherapy is often required to address symptom control and preserve quality of life [20]. The observed increase in antidepressant use among vulnerable participants may reflect improved identification and management of depressive symptoms or a therapeutic shift away from higher-risk sedative medications. Importantly, it also highlights the limitations of relying exclusively on PIM-based metrics as indicators of prescribing quality. Medication appropriateness is a multidimensional construct, and, accordingly, medications classified as potentially inappropriate may still be clinically appropriate in specific situations, when aligned with individual goals and expected benefits.

In contrast to the limited quantitative changes, a small but statistically significant reduction in the mean number of PIMs per participant was observed, with consistent downward trends identified throughout frailty categories. Although modest, these changes may be clinically meaningful, particularly when they pertain to high-risk medication classes. Significant reductions were noted in PIMs affecting the nervous and genitourinary systems, which are commonly linked to falls, cognitive impairment, and other adverse outcomes in older adults. This evidence suggests that the medication review component of the intervention likely contributed to targeting medications with a higher potential for harm, even within a relatively short follow-up period.

The reductions observed in psycholeptics and analgesics may suggest that including medication review with non-pharmacological interventions, such as structured exercise and lifestyle counseling, could facilitate deprescribing of symptom-targeted medications. These medications are highly prevalent and are frequently prescribed by clinicians lacking specialized training in geriatrics. They carry significant potential for harm and are consequently a central focus of geriatric clinical practice [28]. We acknowledge that the observed changes in medication-related outcomes are likely driven mainly by the comprehensive medication review. However, this component is embedded within a broader geriatric assessment and multidisciplinary framework, which allowed individualized decision-making, adherence, and coordination with primary care.

However, the lack of significant changes in anticholinergic burden points to the complexity of optimizing pharmacotherapy in older populations. Medications that add to the anticholinergic load are often long-standing treatments with perceived benefits, and their modification may necessitate longer follow-up periods and repeated review. Previous randomized and observational studies have also reported difficulties in attaining sustained reductions in overall drug burden, highlighting the incremental nature of deprescribing in real-world practice [13, 15, 26].

When stratified by frailty status, trends toward improved medication-related outcomes were observed across all groups, although these changes were generally less pronounced among frail participants. This outcome may reflect clinical reality rather than a failure of intervention. Frail individuals often require ongoing pharmacological treatment to manage complex symptom profiles, acute exacerbations of chronic disease, and palliative care needs [9, 22, 30]. Additionally, while evidence-based deprescribing resources are available, their application in frail and multimorbid populations remains challenging. This aspect may contribute to uncertainty in clinical decision-making, and even therapeutic inertia, especially when medications are not clearly linked to adverse effects [11, 31].

From an implementation perspective, these results provide insights into the incorporation of a geriatric-led medication review into routine, community-based care, highlighting its integration and limitations. The clinically driven medication review reflects the heterogeneity in real-world practice and underscores the importance of person-centered approaches in routine care. This pragmatic design enhances the external validity of the findings, though it also limits the ability to draw causal conclusions.

This study has several strengths, including its real-world setting, the integration of the medication review into a multidimensional intervention, and the stratification by frailty status, which enabled the exploration of prescribing trajectories across varied older populations. However, several limitations should be acknowledged. The absence of a control group prevents causal attribution, and the three-month follow-up may have limited the ability to detect longer-term or cumulative effects. Medication appropriateness was evaluated using PIM lists and anticholinergic burden as proxies, which do not completely represent individual treatment goals or patient-reported outcomes. As such, they do not provide a complete assessment of medication appropriateness, which is a multidimensional construct, encompassing both over- and under-prescription. Additionally, the sample size, mainly within frailty subgroups, may have limited the statistical power to detect smaller but clinically meaningful changes. In particular, the use of a three-level frailty stratification, while clinically meaningful, may have limited statistical power and the ability to detect subtle differences between groups. Finally, a proportion of participants were lost to follow-up, and reasons for attrition were not fully available for all cases. Although reflecting the real-world implementation, from a scientific point of view it may introduce potential attrition bias and should be considered when interpreting the results.

## Conclusions

In conclusion, the + AGIL Barcelona program suggests that a geriatric-led, multidimensional intervention, within an integrated care program, was associated with improvements in qualitative aspects of medication use in community-dwelling older adults, even without substantial reductions in overall medication burden. The data emphasize the importance of prioritizing medication appropriateness over medication count in frailty-informed care. Future research should include longer follow-up periods, patient-centered outcomes, and combined assessments of potentially inappropriate medications and prescribing omissions, along with standardized yet adaptable deprescribing approaches to more effectively capture the longitudinal impact of pharmacotherapy optimization in aging populations.

## Data Availability

All data generated or analyzed during this study are available upon reasonable request to the corresponding author.

## Abbreviations

95%CI: 95% Confidence interval
ACB: Anticholinergic burden
ATC: Anatomical therapeutic clinical
CFS: Clinical frailty scale
CGA: Comprehensive geriatric assessment
OR: Odds ratio
PIM: Potentially inappropriate medication
SD: Standard deviation
